# Treatment outcomes of Spetzler-Ponce grade A brain AVMs in a low-volume center: a systematic review and single institution experience

**DOI:** 10.1007/s10143-025-03991-3

**Published:** 2025-12-10

**Authors:** Patrik Järvelin, Henri Pekonen, Timo Koivisto, Juhana Frösen

**Affiliations:** 1 Hemorrhagic Brain Pathology Research Group, Kuopio University Hospital, Kuopio, Finland; 2 Hemorrhagic Brain Pathology Research Group, Faculty of Medicine and Health Technology, Tampere University, Tampere, Finland; 3 Dept of Neurosurgery, Kuopio University Hospital, Kuopio, Finland; 4 Dept of Neurosurgery and Tays Research Services, Wellbeing Services County of Pirkanmaa, Tampere University Hospital, Tampere, Finland

## Abstract

Arteriovenous malformations of the brain (bAVMs) are complex lesions in the treatment of which patient volumes and subsequent experience are likely to reflect on the outcome. We investigated whether a small neurosurgical center with a limited caseload can achieve similar results to larger centers in treatment of low grade bAVMs. We performed a systematic literature review of studies that have reported outcomes for Spetzler-Ponce A bAVM patients and completed a statistical analysis with a binary random-effects model (Simonian Laird). Since the definition of morbidity varied significantly between studies, we also grouped surgical and endovascular studies according to the definition. We examined treatment outcomes at our own institution and compared them to the literature. In the random effects model based on the literature review, surgery achieved a cure more consistently (98.2%, CI 97.3–99.2%, I^2^ = 50.8) than embolization (71.0%, CI 61.5–80.5%, I^2^ = 97.3) or radiosurgery (77.7%, CI 72.8–82.6%, I^2^ = 90.0). Surgical morbidity (5.4%, CI 3.7–7.1%, I^2^ = 85.3) in the literature was comparable to embolization (2.9%, CI 1.4–4.4%, I^2^ = 43.1) and radiosurgery (6.2%, CI 1.6–10.7%, I^2^ = 77.7). When studies were grouped according to the definition of morbidity, 9.5% experienced a new neurological deficit, 8.7% developed any worsening in mRS and 3.3% severely worsened in mRS after surgery. At our own institution in 1980–2019, 16.9% (14/83) developed any mRS worsening and 6.0% severely worsened in mRS after surgery. Treatment outcomes improved in 2010–2019 (after adoption of radiosurgery) in comparison to earlier decades (*p* = 0.213), with 11.7% (2/17) experiencing any and 0% (0/17) severe mRS worsening after surgery. Radiosurgical cure rate at our institution was 82.1% (23/28). While surgical morbidity at our institution has been somewhat higher than what is reported in the literature, outcomes have improved during the latest decade after adoption of radiosurgery. Radiosurgical outcomes at our institution were comparable to the literature. A small center can treat low grade bAVMs safely with surgery and radiosurgery given adequate training and proper patient selection.

**Supplementary Information:**

The online version contains supplementary material available at 10.1007/s10143-025-03991-3.

## Introduction

Based on the ubiquitously adopted Spetzler-Martin grade [1], the 3-tier Spetzler-Ponce (SP) grade [2] divides arteriovenous malformations of the brain (bAVMs) into three groups which roughly correspond to the operability of the lesion: most SP A bAVMs can be operated on with acceptable surgical risk, while this holds true for only some SP B and few (if any) SP C bAVMs [2]. In addition to the SP grade, hemorrhagic presentation is an important prognostic factor. Ruptured bAVMs are more likely to rupture again [3–6], and the hematoma (once removed) may offer space and a plane of dissection to the bAVM nidus, allowing excision of the bAVM with less additional parenchymal damage in comparison to operating on an unruptured patient.

The randomized, multicenter Aruba-trial [7] found medical management superior to interventional therapy in unruptured bAVMs. ARUBA included surgically, endovascularly and radiosurgically treated patients with several undergoing multiple treatment modalities. Most patients in the intervention group of the trial underwent embolization, and only 44% of the patients in the intervention group experienced cure of their lesion. While the findings of the trial have justifiably risen discussion on whether unruptured bAVMs should be intervened on at all, the relatively short duration of follow-up, low overall cure rate and high ratio of embolized patients in the trial question whether conclusions on the viability of surgery or radiosurgery can be drawn based on the trial. After ARUBA, several case series on comparable patients have been published with acceptable surgical and radiosurgical results [8].

Both Spetzler-Martin [1] as well as the Spetzler-Ponce [2] grading are based on the treatment results of a single quarternary referral center. Given that bAVMs are complex lesions in the treatment of which volumes and subsequent experience are likely to reflect on the outcome, it can be questioned whether the experiences of larger centers can be extrapolated into a smaller center. The aims of this study were to (i) investigate the safety and treatment outcomes of surgery, embolization and radiosurgery in low grade bAVMs and (ii) investigate whether a small center can achieve comparable results by examining our own outcomes.

## Materials and methods

We performed a systematic literature review according to the Preferred Reporting Items for Systematic Reviews and Meta-Analyses (PRISMA) guidelines [9] and compared that with our own, single institution clinical series.

### Systematic literature review

A Pubmed-database search was performed for entries up until December 2023 using the following query guidelines: “(arteriovenous malformation OR AVM) AND brain AND surgery”, “(arteriovenous malformation OR AVM) AND brain AND (endovascular OR onyx OR embolization)” and “(arteriovenous malformation OR AVM) AND radiosurgery”. Additionally, the clinical trials registry (clinicaltrials.gov) was searched for trials with the query “AVM” up until December 2023. The relevance of the retrieved articles was assessed by reading the titles, abstracts or both.

**Fig. 1:**
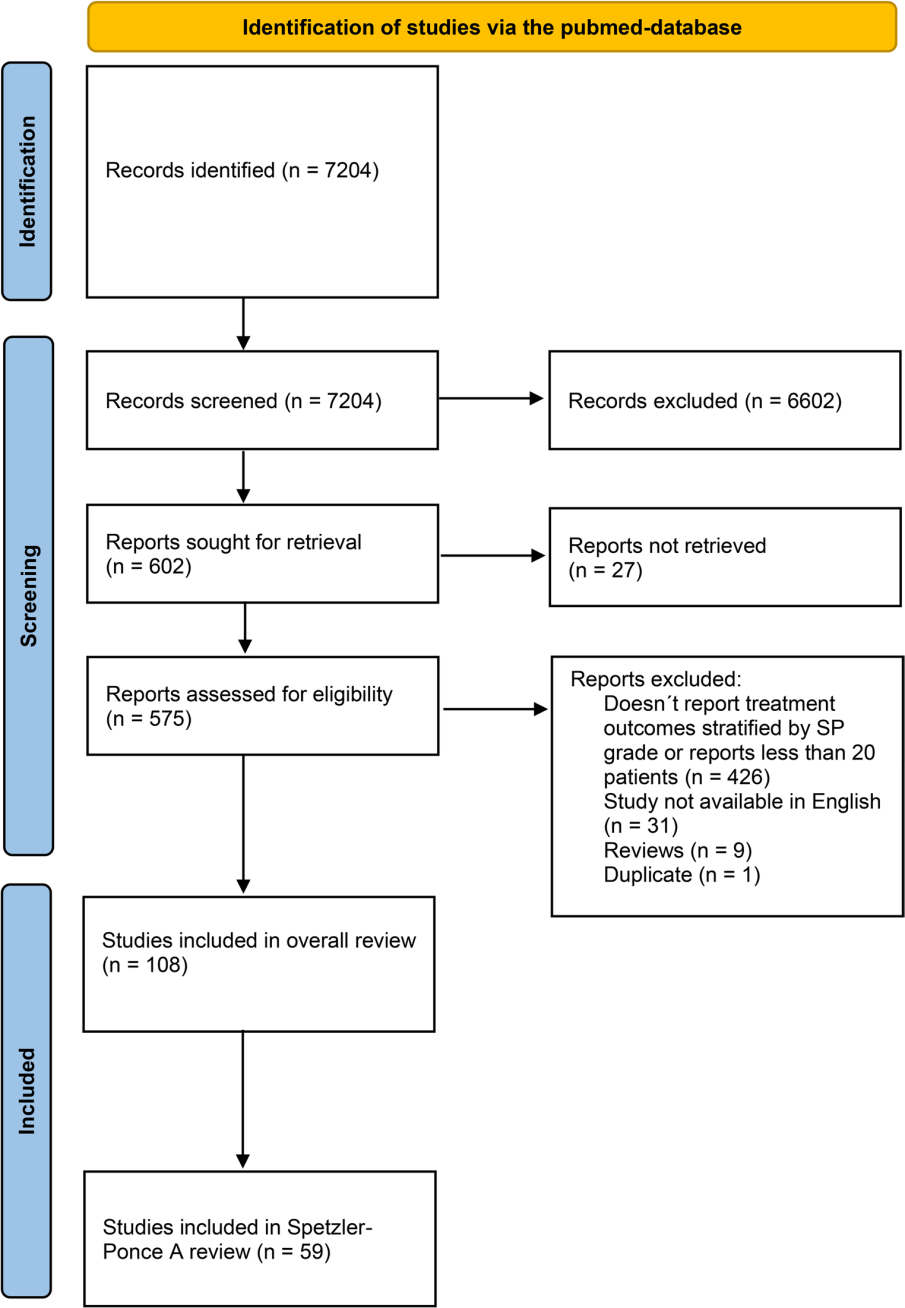
Flow diagram illustrating the review process

Inclusion criteria required that each article is a peer-reviewed clinical study or a case series of bAVMs treated with surgery, endovascular embolization or radiosurgery written in English. Each treatment modality was assessed separately. In addition, inclusion criteria for surgical studies require that surgical morbidity was reported. For radiosurgical and endovascular studies, the inclusion criteria require that cure rates were reported. Case volume of the reporting institution was assessed: if information wasn´t provided in the manuscript, corresponding authors were contacted with email to determine the case volume of the institution. The literature review was initially performed for all three Spetzler-Ponce subgroups, but for the purposes of this review studies which did not report outcomes for Spetzler-Ponce A bAVMs were later excluded. Since very small cohorts from a single institution are likely to include considerable variance based on the small sample size, studies which reported less than 20 patients were excluded. These studies are referenced in the Data Supplement (Table S1). Studies in which treatment did not aim for a complete cure were excluded. To avoid overlapping cohorts, when several publications originating from the center, era and patient population were eligible for inclusion the larger study was chosen. Studies were cross-referenced for suitable series. The review process is illustrated in Fig. 1. The review wasn´t registered and a protocol wasn´t prepared.

The inclusion criteria were applied to the search results and relevant biases assessed by the first author (P.J.). Data extraction was performed as per individual study by the first author (P.J.). The procedure-associated morbidity, mortality and rate of complete occlusion of the lesion were used as outcome measures. Studies which didn´t provide information for an outcome measure were excluded from the related synthesis. The definition of morbidity in the reviewed study was used when assessing each individual study. Since the definition of morbidity used in studies varied, a subgroup analysis for surgical and endovascular studies was performed based on the definition. Morbidity from preoperative embolization prior to surgery was included in surgical morbidity. For radiosurgery, morbidity from a bAVM rupture during the latency period after treatment was classified under procedure-associated morbidity. Rate of occlusion of the lesion was assessed with either DSA or MRA.

Statistics were calculated with SPSS 22.0 (IBM, Armonik, NY) software. Due to high variations in study characteristics, a statistical analysis using a binary random-effects model (DerSimonian-Laird method) was performed using OpenMeta[analyst] software (Agency for Healthcare Research and Quality). The OpenMeta[analyst] software was used to create forest plots which present the literature review. Outcomes across studies studies were pooled using a random-effects model and reported with a 95% confidence interval (CI). Heterogeneity was assessed with Cochran´s *Q* test and *I*^*2*^ statistic. SPSS, Microsoft Word and Excel were used to tabulate and visually display results on individual studies and syntheses.

### Single institution clinical series from Kuopio university hospital

This is a non-blinded, retrospective series based on the Kuopio University Hospital (KUH) bAVM registry. The KUH bAVM registry was created by a systematic search of the KUH hospital discharge registry for ICD-10 diagnosis codes Q28.0–28.3.3, complemented by a retrospective review of operating room logbooks ranging to 1981. bAVM patients from 1981 to 2019 were included. Following this, the medical records of all identified patients were reviewed. Confirmed bAVM cases were entered to the registry, and for them the date of diagnosis, age at diagnosis, sex and neurological symptoms described in the patient´s medical records at the time of diagnosis or leading to diagnosis were recorded. Additionally, the treatments given, dates of interventions and postoperative neurological condition were recorded. Posttreatment clinical outcome was assessed according to modified Rankin Scale (mRS). For this study, we collected all SP A patients from the KUH bAVM registry. Morbidity, mortality and cure rate were used as outcome measures.

BAVM patients in KUH are treated by a multidisciplinary team consisting of neurosurgeons and radiologists. At a given time there is a single, senior surgeon responsible for surgical treatment of bAVMs. Despite this centralization to a single surgeon, given the low number of operated cases annually, experience on bAVM surgery has accumulated gradually over a long period of time during which the primary bAVM surgeon has changed three times. There is another surgeon responsible for radiosurgery of bAVMs, which has also changed three times. Treatment plans are formed in a multidisciplinary meeting consisting of neurovascular surgeons and interventional radiologists.

Statistics were calculated with SPSS 22.0 (IBM, Armonik, NY) software. For continuous variables, median and range were calculated and Mann-Whitney U-test used for comparison. For categorical variables, proportions and percentages were calculated and Chi-Square test used for statistical comparison.

## Results

Including the present study, 26 surgical, 14 endovascular and 22 radiosurgical publications were included in the literature review. We also looked at surgical and radiosurgical treatment outcomes at our institution and compared them to the literature. Patients were not treated with stand-alone embolization at our institution. Radiosurgery became available in our institution as a treatment modality in the 2000s: in 2000–2009 31% (8/26) and in 2010–2019 54% (20/37) of SP A bAVMs in KUH were treated with radiosurgery.

### Treatment outcomes according to the systematic literature review

The reviewed studies included 2616 surgical, 1075 endovascular and 2741 radiosurgical SP A bAVMs. Surgery achieved a superior rate of occlusion (mean 97.4%, median 97.7%, interquartile range 96.0–100.0%) in comparison to endovascular embolization (mean 70.0%, median 72.0%, interquartile range 45.5–93.2%) and radiosurgery (mean 77.8%, median 79.6%, interquartile range 74.3–89.2%). Morbidity was not significantly different between treatment methods: mean 7.3% (median 4.2%, interquartile range 1.7–10.7%) for surgery, 3.7% (median 2.7%, interquartile range 0.5–6.6%) for endovascular embolization and 7.1% (median 6.0%, interquartile range 1.7–2.7%) for radiosurgery. When surgical studies were divided into groups based on the definition of morbidity, average surgical morbidity was 9.5% for studies which defined morbidity as a new neurological deficit, 8.7% for studies which defined morbidity as an increase in mRS and 3.3% in studies which defined morbidity as an outcome of mRS > 1 or > 2. Mortality was comparable between treatment methods: 0.5% (median 0%, interquartile range 0–0.1.1%) for surgery, 0.2% (median 0%, interquartile range 0–0.4.4%) for embolization and 0.9% (median 0%, interquartile range 0–2.3.3%) for radiosurgery. The literature review is presented in Tables 1, 2 and 3. Forest plots are provided in Figures S1−3 (Data Supplement).

**Table 1:** Literature review on surgical treatment of Spetzler-Ponce A bAVMs. The table is divided into three groups based on morbidity definitions: average morbidity, mortality and rate of occlusion for each section is shown under the group. The averages for the entire table are at the bottom

Study	Number of patients (*n* = 2616)	Morbidity	Definition of morbidity	Mortality	Rate of occlusion
Hartmann et al. [10] (2000)	48	29.2%	Neurological deficit	0%	N/A
Teo et al. [11] (2016)	48	10.4%	Neurological deficit	0%	N/A
Wong et al. [12] (2017)	118	10.2%	Neurological deficit	N/A	96.6%
Tong et al. [13] (2017)	186	0%	Neurological deficit	0%	100%
Aboukaïs et al. [14] (2017)	64	0%	Neurological deficit	N/A	98.4%
Schramm et al. [15] (2017)	167	7.2%	Neurological deficit	0%	N/A
Chen et al. [16] (2019)	41	24.4%	Neurological deficit	N/A	100%
Karki et al. [17] (2021)	21	19%	Neurological deficit	0%	N/A
Maalim et al. [18] (2023)	111	2.7%	Neurological deficit	N/A	N/A
Krivoshapkin et al. [19] (2005)	22	0%	Mild or major neurological deficit	0%	100%
Spetzler et al. [1] (1986)	44	0%	Major neurological deficit	0%	N/A
Gross et al. [20] (2015)	44	11.4%	Neurological complication	N/A	N/A
On average		***9.5%***		***0%***	***99%***
Javadpour et al. [21] (2016)	24	8.3%	Neurological deficit with deterioration in mRS	0%	100%
Gorgan et al. [22] (2020)	43	0%	Increased mRS	0%	97.7%
Sai Kiran et al. [23] (2020)	27	3.7%	Increased mRS	3.7%	96.3%
Gami et al. [24] (2021)	113	13%	Increased mRS	N/A	96%
Steinberg et al. [25] (2021)	65	3.1%	Increased mRS	N/A	89.2%
Catapano et al. [26] (2022)	406	18.2%	Increased mRS	N/A	N/A
Al-Smadi et al. [27] (2019)	34	5.9%	Increased mRS	0%	N/A
On average		***8.7%***		***0.9%***	***95.8%***
Patel et al. [28] (2019)	110	1.8%	Neurological deficit with mRS > 1	N/A	95%
Darsaut et al. [29] (2022)	110	2.7%	Increased mRS, final mRS atleast > 2	N/A	95.5%
Potts et al. [30] (2015)	232	3%	Increased mRS, final mRS atleast > = 2	0.4%	100%
Järvelin et al. (2025)	83	6.0%	Increased mRS, >=2	2.4%	96.3%
Korja et al. [31] (2014)	361	1.4%	mRS > 1	N/A	N/A
Steiger et al. [32] (2015)	69	4.3%	mRS > 1	N/A	100%
Weber et al. [33] (2007)	25	4%	mRS > = 2	0%	N/A
On average		***3.3%***		***0.9%***	***97.4%***
On average (total)		**7.3%**		**0.5%**	**97.4%**

**Table 2:** Literature review on endovascular embolization of Spetzler-Ponce A bAVMs. The table is divided into three groups based on morbidity definitions: average morbidity, mortality and rate of occlusion for each section is shown under the group. The averages for the entire table are at the bottom

Study	Number of patients (*n* = 1075)	Morbidity	Definition of morbidity	Mortality	Rate of occlusion
Saatci et al. [34] (2011)	158	2.5%	Neurological deficit	0.6%	98.1%
Baharvahdat et al. [35] (2019)	224	5.4%	Neurological deficit	0.4%	91.5%
Razavi et al. [36] (2022)	109	0.9%	Neurological deficit	0%	89.9%
Poncyljusz et al. [37] (2017)	24	8.3%	Neurological deficit	0%	79.2%
On average		***4.3%***		***0.3%***	***89.7%***
Liu et al. [38] (2014)	31	0%	Disabling neurological deficit	0%	87.1%
Willinsky et al. [39] (2001)	50	0%	Major morbidity	0%	38%
Iosif et al. [40] (2019)	73	2.7%	Procedure-related morbidity	0%	98.6%
Raymond et al. [41] (2022)	66	7.6%	Increased mRS, at least > 2	0%	34.8%
On average		***2.6%***		***0%***	***64.6%***
Talaat et al. [42] (2022)	145	5.5%	Increased mRS	0.7%	37.9%
Van Rooij et al. [43] (2012)	20	N/A	N/A	N/A	100%
Strauss et al. [44] (2013)	25	N/A	N/A	0%	48%
Consoli et al. [45] (2014)	40	N/A	N/A	N/A	50%
Rodriguez-Calienes et al. [46] (2022)	42	N/A	N/A	N/A	61.9%
Rodriguez-Calienes et al. [47] (2023)	68	N/A	N/A	0%	64.7%
On average		**3.7%**		**0.2%**	**70.0%**

**Table 3:** Literature review on radiosurgery of Spetzler-Ponce A bAVMs

Study	Number of patients (*n* = 2741)	Morbidity	Mortality	Rate of occlusion
Friedman et al. [48] (2003)	107	N/A	N/A	75.5%
Andrade-Souza et al. [49] (2005)	55	N/A	N/A	78.2%
Reyns et al. [50] (2007)	45	2.2%	N/A	88.9%
Kiran et al. [51] (2007)	20	N/A	N/A	90%
Kano et al. [52] (2012)	217	N/A	2.8%	90%
Milker-Zabel et al. [53] (2012)	169	N/A	N/A	47.3%
Koltz et al. [54] (2013)	33	N/A	0%	90.9%
Ding et al. [55] (2014)	502	5.6%	N/A	76.1%
Nicolato et al. [56] (2015)	34	N/A	N/A	79.4%
Boström et al. [57] (2016)	32	6.3%	0%	80.6%
Ding et al. [58] (2017)	232	11.2%	1.7%	79.7%
Thenier-Villa et al. [59] (2017)	87	N/A	N/A	92.0%
Raboud et al. [60] (2018)	23	N/A	N/A	87.0%
Hasegawa et al. [61] (2019)	86	N/A	N/A	66%
Tuleasca et al. [62] (2020)	113	N/A	N/A	70.8%
Gami et al. [24] (2021)	120	17%	N/A	57%
Erickson et al. [63] (2022)	89	N/A	N/A	51.7%
Nguyen et al. [64] (2022)	413	N/A	N/A	75.5%
Hak et al. [65] (2022)	153	N/A	N/A	77.8%
Bethanabatla et al. [66] (2022)	103	N/A	N/A	84.5%
Sasagasako et al. [67] (2023)	80	N/A	N/A	90%
Järvelin et al. (2025)	28	0%	0%	82.1%
On average		**7.1%**	**0.9%**	**77.8%**

## Random effects model

In the random effects model, surgery was associated with a morbidity of 5.4% (CI 3.7–7.1%, I^2^ = 85.3), mortality of 0.5% (CI 0.1–0.9%, I^2^ = 0) and cure rate of 98.2% (CI 97.3–99.2%, I^2^ = 50.8). Embolization was associated with a morbidity of 2.9% (CI 1.4–4.4%, I^2^ = 43.1), mortality of 0.6% (CI 0.1–1.1%, I^2^ = 0) and cure rate of 71.0% (CI 61.5–80.5%, I^2^ = 97.3), while radiosurgery was associated with a morbidity of 6.2% (CI 1.6–10.7%, I^2^ = 77.7), mortality of 2.0% (CI 0.8–3.2%, I^2^ = 0) and cure rate of 77.7% (CI 72.8–82.6%, I^2^ = 90.0).

### Impact of volume on the treatment results

We investigated the effect of institutional case-volume on the treatment results by plotting the procedure-associated morbidity or cure rate reported in the literature against the case-volume of the corresponding center as reported in the manuscript or through an email query (Data Supplement, Figures S4-6). We did not find a statistically significant difference in surgical morbidity or endovascular and radiosurgical cure rates between smaller and larger centers, although there was a trend of higher institutional patient volume leading to lower surgical morbidity. Surgical morbidity had considerable variance which is partly explained by differences in the definition of morbidity in included studies. Endovascular cure rates had similar variance regardless of institutional volume. In comparison, radiosurgery achieved consistent results across reporting institutions.

## Surgical outcomes in KUH

The surgical cohort in KUH included 83 SP A bAVMs. In 1981–2019, 16.9% (14/83) of surgical patients experienced any and 6.0% (5/83) severe treatment-related worsening of mRS. Severe worsening or mRS was defined as worsened mRS with a final mRS of 2 or higher. Complications from preoperative embolization were included in surgical morbidity: 3 out of 5 cases of severe worsening of mRS were related to preoperative embolization. Surgery had an angiographically confirmed cure rate of 95.2% (79/83). One patient died after postsurgery hemorrhage and another died to complications of preoperative embolization accounting for a surgical mortality of 2.4% (2/83). Surgical outcomes improved in 2010–2019 in comparison to earlier decades (*p* = 0.213), with 11.7% (2/17) of surgical patients in 2010–2019 experiencing any and 0% (0/17) severe treatment-related worsening of mRS. The overall outcomes in 1981–2019 are shown in Table 4, while outcomes by decade are shown in Table 5.

**Table 4:** Treatment results for Spetzler-Ponce A bAVMs in Kuopio university hospital stratified by treatment method

	Rate of occlusion	Treatment-associated increase in mRS	Treatment-associated increase in mRS, final mRS > = 2	Mortality	mRS
Only surgery	98.0% (48/49)	14.3% (7/49)	4.1% (2/49)	2.0% (1/49)	0.78
Pre-operative embolization and surgery*	93.9% (31/33)	21.2% (7/33)	9.1% (3/33)	3.0% (1/33)	1.03
All surgical patients	95.2% (79/83)	16.9% (14/83)	6.0% (5/83)	2.4% (2/83)	0.87
Only embolization	0% (0/1)	0% (0/1)	0% (0/1)	0% (0/1)	0
Surgery into radiosurgery	100% (1/1)	0% (0/1)	0% (0/1)	0% (0/1)	0
Pre-operative embolization and radiosurgery	80.0% (4/5)	20.0% (1/5)	0% (0/5)	0% (0/4)	1
Radiosurgery	81.8% (18/22)	0% (0/22)	0% (0/22)	0% (0/22)	0.41
All radiosurgical patients	82.1% (23/28)	3.6% (1/28)	0% (0/28)	0% (0/28)	0.50

*Includes one patient who died to complications of preoperative embolization

**Table 5:** Surgical treatment outcomes in Kuopio university hospital stratified by decade

	Number of surgically treated bAVMs (number of preoperatively embolized patients in brackets)	Treatment-associated increase in mRS	Treatment-associated increase in mRS, final mRS >=	Mortality	Occlusion
2010s	17 (8 embolized)	11.8% (2/17)	0% (0/17)	0% (0/17)	94.1% (16/17)
2000s	18 (10 embolized)	22.2% (4/18)	11.1% (2/18)	5.6% (1/18)	88.9% (16/18)
1990s	34 (15 embolized)	14.7% (5/34)	5.9% (2/34)	2.9% (1/34)	97.1% (34/35)
1980s	14 (none embolized)	21.4% (3/14)	7.1% (1/14)	0% (0/14)	100% (14/14)

33/83 (39.8%) of surgical patients underwent preoperative embolization prior to surgery. No embolizations were done during the first decade from 1980 to 1989. In 1990–2019, 33/69 (47.8%) of patients were preoperatively embolized before surgery. This ratio has been consistent during the decades. The KUH patient demographics are presented in Table 6.

**Table 6:** Demographics of Spetzler-Ponce A bAVM patients in Kuopio university hospital

Age (min-max, median)	8–79 (38)
Sex (% of females)	35.1% (39/111)
Presentation with hemorrhage	56.8% (63/111)
Focal neurological deficit	36.9% (41/111)
Motor deficit	18.0% (20/111)
Sensory deficit	7.2% (8/111)
Visual field deficit	8.1% (9/111)
Cognitive impairment	4.5% (5/111)
Epilepsy	31.5% (35/111)

## Radiosurgical outcomes in KUH

The radiosurgical cohort in KUH includes 28 SP A bAVMs, 5 (18%) of which underwent embolization prior to radiosurgery. 2 patients initially had their bAVM surgically excised and later received either standalone radiosurgery or embolization combined with radiosurgery for a recurrent lesion. One patient underwent partial surgery prior to radiosurgery for a residive lesion. The KUH patient demographics are presented in Table 6.

**Fig. 2:**
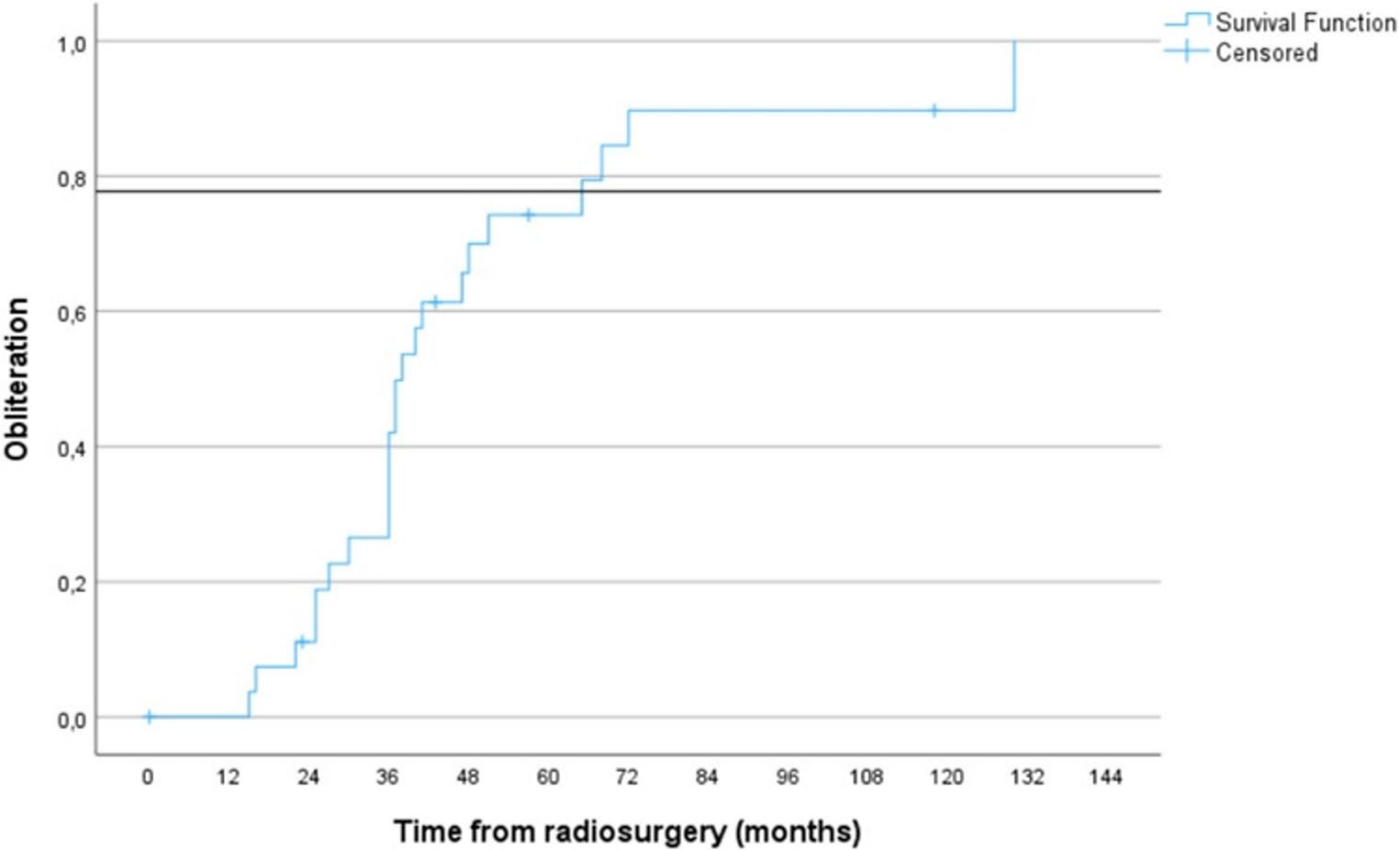
A Kaplan-Meier graph in which occurrence of a cure after radiosurgery was used as the endpoint. The y-axis represents time which has passed since radiosurgery. This figure demonstrates that most radiosurgically treated SP A bAVMs achieve a cure approximately 2–4 years after treatment. The horizontal line represents average obliteration rate in the literature, derived from the random effects model

Radiologic imaging changes (RICs) related to radiosurgery developed in 7% (2/28): these were asymptomatic in one patient and symptomatic but transient in one patient. 1 patient developed ataxia of the left hand 4 years after treatment. His imaging showed signs of minor, new intracranial hemorrhage in the region of the bAVM despite DSA confirming absence of the lesion: this was the only posttreatment hemorrhage in our cohort. Overall, there was no severe increase in mRS or mortality after radiosurgery. Radiosurgery had an overall cure rate of 82.1% (23/28) and an actuarial cure rate of 28.6%) (8/28) at 3 years, 67.9% (19/28) at 5 years and 78.6% (22/28) at 7 years after radiosurgery. On average, obliteration of the lesion was achieved 3.5 years from initial radiosurgery (median 3.1 years, range 1.3–10.3.8y). Moreover, out of the 5 SP A bAVM patients whose lesion did not occlude after radiosurgery, two patients died of lung cancer, one moved away from our hospital network and two were purposefully lost to follow-up, one due to old age and the other due to low functional independency related to bAVM hemorrhage prior to radiosurgical treatment. Outside these 5 patients with a short follow-up due to circumstances unrelated to radiosurgery, all radiosurgically treated SP A bAVMs in KUH eventually occluded. A Kaplan-Meier plot illustrating the occurrence of a cure as a function of time is provided in Fig. 2. The KUH outcomes are presented in Table 4.

## Discussion

Surgery for Spetzler-Ponce A bAVMs is considered safe and has been validated by several series (Table 1), although most of these originate from larger centers. Achieving patient volumes comparable to the large referral centers is not feasible in many smaller countries, and it is uncertain whether the widely cited findings of large centers can be extrapolated to smaller centers. Additionally, embolization and radiosurgery may also yield good results. We compared treatment outcomes of different modalities in a literature review and investigated our own outcomes to assess how SP A bAVMs should be treated and whether treatment can be safe in a smaller center.

We found surgery to be the most reliable (cure rate of 98.2% in the random effects model) and comparably safe (morbidity rate of 5.4% in the random effects model) treatment modality. Since the anatomy of a SP A bAVM is considered favorable and subsequent surgical risk relatively low, surgery can instantaneously remove the risk of rupture with acceptable risk in most patients. While radiosurgery was comparably safe (morbidity rate of 6.2% in the random effects model), it was less reliable (cure rate of 77.7%% in the random effects model). Embolization was also safe (morbidity rate of 2.9% in the random effects model), although interestingly there was significant variance in cure rates between different series (cure rate of 71.0% in the random effects model, minimum 34.8%, maximum 100%). This likely reflects differences in endovascular technique and introduction of newer endovascular techniques in addition to a possibly high dependence of the results on the interventionalist´s expertise.

While the radiosurgical results of our own institution were comparable to the literature, surgical morbidity in our institution´s history has been somewhat higher than in the literature: 16.9% of our patients experienced worsening in mRS after surgery in comparison to 8.7% in the literature. Correspondingly, more severe deficits were also more common with 6.0% of patients in our institution in comparison to 3.3% in the literature experiencing severe worsening in mRS. It is notable that outcomes have improved in the recent decade, as patients treated at our institution in the 2010 s have done better than patients who were treated earlier. This likely reflects acquired surgical expertise and, most importantly, improved patient selection as radiosurgery has become widely used at our institution during the latest decade. In the 2010 s, more than half (20/37) of treated SP A bAVMs underwent radiosurgery. This is a significant difference to the 2000 s (8/26 underwent radiosurgery) and especially the 1980–90s during which radiosurgery was not available at our institution. In KUH, treatment plans for bAVM patients are discussed in a multidisciplinary meeting consisting of neurovascular surgeons and interventional radiologists. Since the physicians responsible for surgery, radiosurgery and embolization in our institution are different people, this meeting allows for discussion on different treatment options and likely avoids personal bias of the treating physician towards their primary treatment of choice. This practice has led to the increased usage of radiosurgery and likely contributed to the improved patient outcomes. Since the treatment outcomes of radiosurgery do not appear to be related to patient volumes, likely even low volume centers should consider training some of their staff on bAVM radiosurgery. BAVM radiosurgery can be performed with a regular LINAC machine [59].

Given that only complete obliteration of a bAVM eliminates the risk of rupture, surgery should be considered the primary course of treatment for young and healthy patients with accessible lesions. Still, our findings and experience suggest that SP A bAVM patients benefit from individual assessment irrespective of the deemed low grade of the lesion. Abstaining from surgery may be beneficial for erderly patients or patients with greater perceived surgical risk, even in low grade bAVMs. Radiosurgery should be considered the primary option for these patients, although embolization may also be considered in centers highly proficient with the treatment modality.

### Limitations

Our own cohort and the series included in the literature review likely include selection bias to varying degrees. The literature review spans over several decades of advancements in treatment and the treatment methods and protocols have progressed over the years. Due to the advancements and institutional practices, treatment protocols were not always comparable between different series. There was considerable variety on how treatment results were reported between different series, especially when it comes to morbidity: the definition of morbidity in a given study greatly influences the reported outcome. For surgical series, this is illustrated in Figure S7 (Data Supplement). Our own patient cohort was relatively small, diminishing the statistical power of our findings.

## Conclusions

Surgery is the most reliable treatment modality and should be considered the golden standard of treatment of SP A bAVMs with accessible lesions and no additional surgical risk factors. Radiosurgery is a great alternative for other patients. Endovascular embolization may achieve comparable results, but outcomes appear to be highly dependent on the individual enter. A small center with adequate training and proper patient selection may achieve safe outomes in treatment of low grade bAVMs. A comprehensive multicenter registry should be conducted to improve the quality of evidence on bAVM outcomes.

## Supplementary Information

Below is the link to the electronic supplementary material.

ESM 1 DOCX (349 KB)

## Data Availability

Following the GDPR act of the European Union, the data from KUH cannot be made publicly available. The data can be provided to established clinical scientists based on a research plan formally approved by the Kuopio University Hospital. Data for the literature review can be provided on request.
