# Supplementary material for: Treatment outcomes of Spetzler-Ponce grade A brain AVMs in a low-volume center: a systematic review and single institution experience
Source: Neurosurg Rev. 2025 Dec 10;49(1):57. doi: 10.1007/s10143-025-03991-3 (PMC12689658; doi:10.1007/s10143-025-03991-3)

Data Supplement

Table S1

Studies which were excluded from the literature review due to having less than 20 patients.

|  | **Number of patients** | **Morbidity** | **Definition of morbidity** | **Mortality** | **Rate of occlusion** |
| --- | --- | --- | --- | --- | --- |
| ***Surgery*** |  |  |  |  |  |
| Santin et al.^68^ (2020) | 6 | 0% | mRS worsening, final mRS >=2 | 0% | 83.3% |
| Kiriş et al.^69^ (2005) | 13 | 7.7% | New neurological deficit | 0% | 92.3% |
| Natarajan et al.^70^ (2008) | 13 | 0% | New neurological deficit | 0% | N/A |
| Cho et al.^71^ (2020) | 8 | 0% | New neurological deficit | 0% | 100% |
| ***On average*** |  | ***1.9%*** |  | ***0%*** | ***91.9%*** |
| ***Embolization*** |  |  |  |  |  |
| Dumont et al.^72^ (2015) | 19 | 0% | New neurological deficit | 0% | 10.5% |
| Alias et al.^73^ (2021) | 15 | 0% | New neurological deficit | 0% | 6.7% |
| Xu et al.^74^ (2019) | 8 | 0% | mRS worsening | 0% | 62.5% |
| Sorimachi et al.^75^ (1999) | 12 | N/A | N/A | N/A | 8.3% |
| van Rooij et al.^76^ (2007) | 15 | N/A | N/A | N/A | 46.7% |
| Pan et al.^77^ (2009) | 9 | N/A | N/A | N/A | 55.6% |
| Tamure et al.^78^ (2015) | 13 | N/A | N/A | N/A | 38.5% |
| de Castro-Afonso et al.^79^ (2016) | 7 | N/A | N/A | N/A | 85.7% |
| ***On average*** |  | ***0%*** |  |  | ***39.3%*** |
| ***Radiosurgery*** |  |  |  |  |  |
| Yamamoto et al.^80^ (1996) | 19 | N/A | N/A | N/A | 63.2% |
| Buis et al.^81^ (2008) | 15 | N/A | N/A | N/A | 80% |

Figure S1

A forest plot demonstrating surgical morbidity for SP A bAVMs in the meta-analysis. The studies included in the meta-analysis are described on the left: 95% confidence intervals for each study as determined by the random effects model are provided. Ev/Trt, events/treatments.


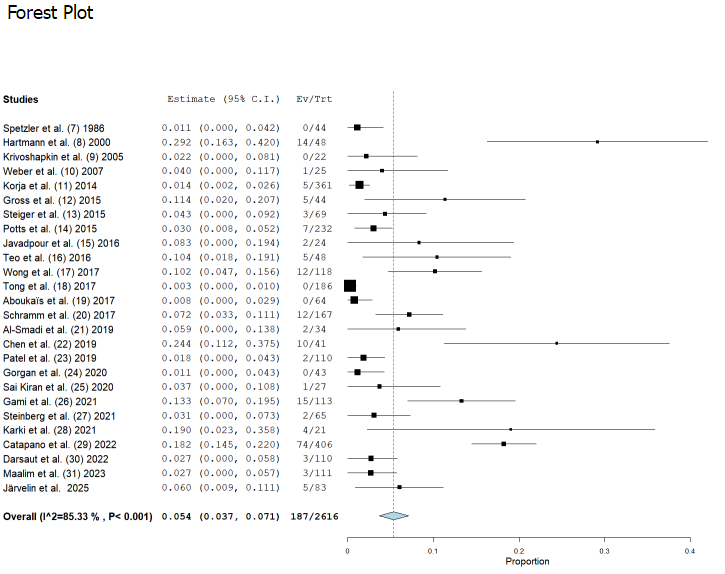


Figure S2

A forest plot demonstrating endovascular cure rates for SP A bAVMs in the meta-analysis. The studies included in the meta-analysis are described on the left: 95% confidence intervals for each study as determined by the random effects model are provided. Ev/Trt, events/treatments.


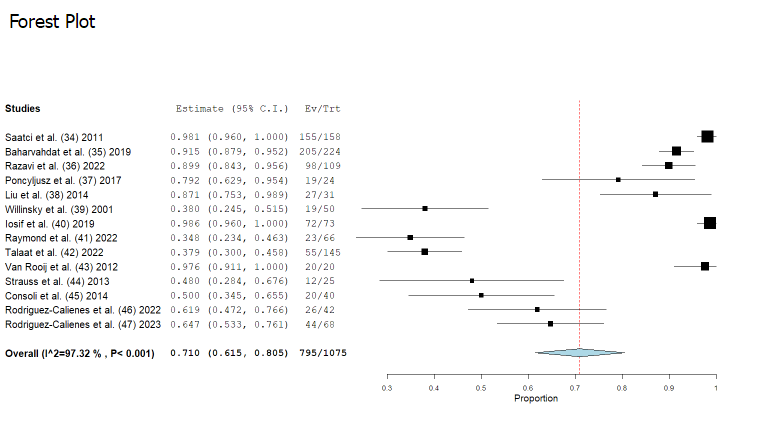


Figure S3

A forest plot demonstrating radiosurgical cure rates for SP A bAVMs in the meta-analysis. The studies included in the meta-analysis are described on the left: 95% confidence intervals for each study as determined by the random effects model are provided. Ev/Trt, events/treatments.


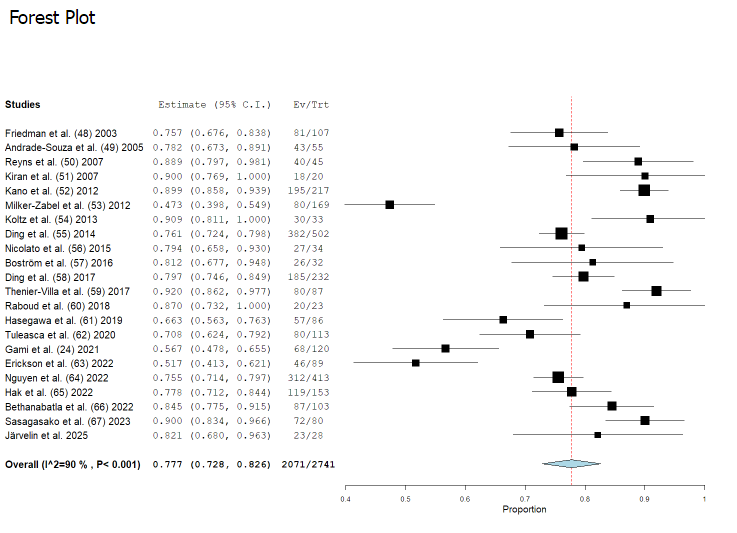


Figure S4

Comparison of surgical morbidity and case volume of the reporting institution. Morbidity is presented on the y-axis and annual case-volume (patient/year) on the x-axis. The dots represent individual studies. Kuopio University Hospital is presented by two dots: the orange dot represents morbidity defined as any treatment related worsening in mRS and the purple dot morbidity defined as worsened mRS with a final mRS of 2 or higher.

Figure S5

Comparison of endovascular cure rates and case volume of the reporting institution. Morbidity is presented on the y-axis and annual case-volume (patient/year) on the x-axis. The dots represent individual studies.

Figure S6

Comparison of radiosurgical cure rates and case volume of the reporting institution. Morbidity is presented on the y-axis and annual case-volume (patient/year) on the x-axis. The dots represent individual studies. The cure rate of KUH is presented by an orange dot.

Figure S7

A visual presentation of surgical morbidity in the literature review. The green bar present studies with a morbidity definition of “neurological deficit”, the orange studies with a definition of “increased mRS” and purple studies with a definition of “(increased) mRS >1 or >2”.


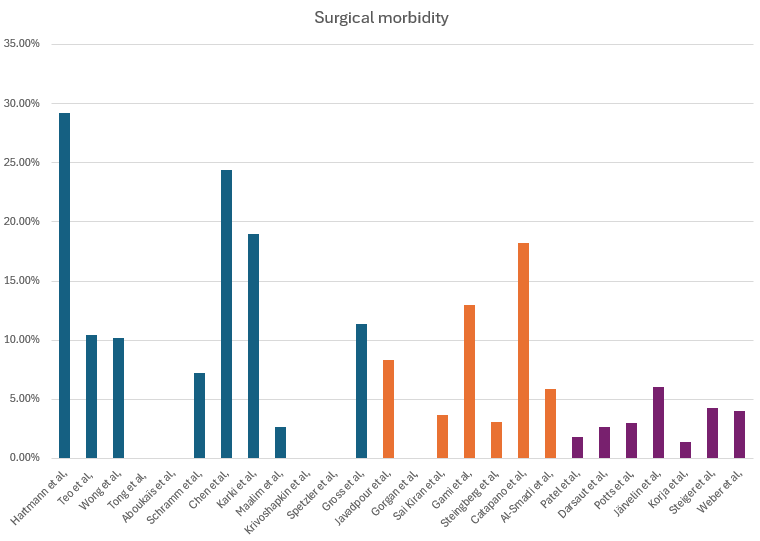


Figure S8

A visual presentation of surgical cure rates in the literature review.


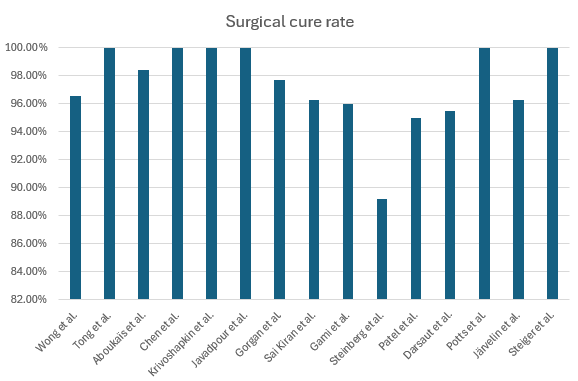


Figure S9

A visual presentation of endovascular morbidity in the literature review.


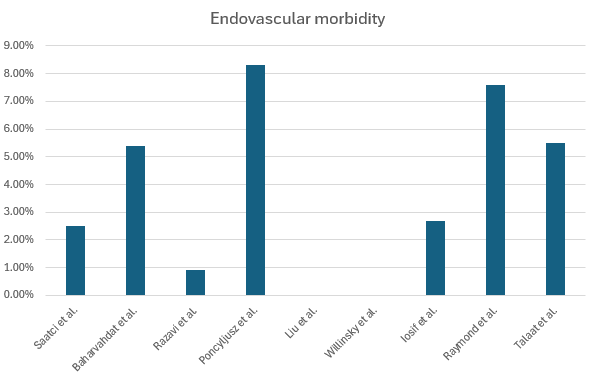


Figure S10

A visual presentation of radiosurgical morbidity in the literature review.


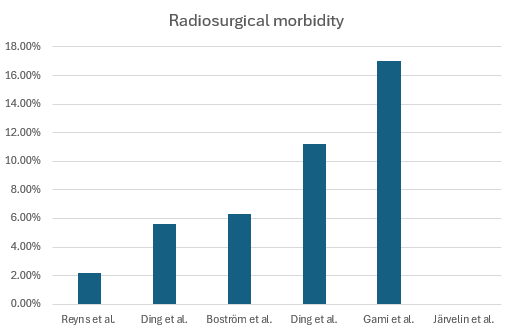

Supplement: Supplementary file 1 — DOCX (349 KB) [file 10143_2025_3991_MOESM1_ESM.docx]
